# Suicides and High School Program Types in Japan

**DOI:** 10.1001/jamanetworkopen.2026.14997

**Published:** 2026-05-27

**Authors:** Rinko Goto, Yuka Nishina, Taro Uchida, Norisuke Kashima, Yusuke Yagai, Kana Ninomiya, Yuya Iijima, Mayumi Hangai

**Affiliations:** 1 Japan Suicide Countermeasures Promotion Center, Tokyo, Japan; 2 Graduate School of Human Sciences, Sophia University, Tokyo, Japan; 3 Faculty of Humanities, University of Toyama, Toyama, Japan

## Abstract

This cross-sectional study compares suicide mortality rates and the characteristics between part-time and correspondence high school programs vs full-time high school programs in Japan.

## Introduction

Suicides among high school students in Japan reached a record high in 2022 and remained elevated in 2024, despite a decreasing student population.^1^ Recently, enrollment in part-time and correspondence (PT/C) high school programs, often attended by students with prior school-related difficulties, including absenteeism, interpersonal problems, mental health issues, and other nontraditional educational needs, has increased.^2^ In Japan, PT/C programs feature flexible formats, including evening or shortened-hour classes designed mainly for working students (part time) or primarily home-based, self-directed remote study (correspondence), whereas full-time (FT) programs represent conventional daytime schooling. Since 2022, national suicide statistics have included a new classification by high school program type, including FT and PT/C programs, enabling comparisons between these groups.^3^ Although part-time programs and correspondence programs differ in instructional format, they are combined into a single PT/C category in the current suicide statistics. This study aimed to compare suicide mortality rates and the characteristics between PT/C and FT programs.

## Methods

This cross-sectional study, which followed the STROBE reporting guideline, analyzed suicide deaths among Japanese high school students in FT and PT/C programs from January 1, 2022, to December 31, 2024.^1^ The research ethics committee of the Japan Suicide Countermeasures Promotion Center approved the study; informed consent was not required because suicide data were anonymized and student population data were publicly available. Students in special-needs schools or schools classified as “other” were excluded because of small sample sizes and limited comparability. Suicide mortality rates per 100 000 students were compared between the FT and PT/C groups using Poisson regression with the log of student population as an offset to estimate incidence rate ratios. The characteristics of students in suicide cases were compared using unadjusted and multivariable logistic regression to estimate odds ratios (ORs); the latter were adjusted for all variables and year simultaneously. For both Poisson and logistic regression models, 2-sided 95% CIs were estimated using the profile likelihood method. Estimates were considered statistically significant if their 2-sided 95% CIs excluded 1.00. Analyses were conducted using R, version 4.4.3.

## Results

This study included 1008 suicide cases (FT, 680 [67.5%]; PT/C, 328 [32.5%]; 528 male [52.4%] and 480 female [47.6%]; mean [SD] age, 16.8 [1.3] years in FT and 17.2 [1.6] years in PT/C) (Table 1). Although the overall number of suicides in the FT group decreased, those in the PT/C group increased. The PT/C group had significantly higher suicide mortality rates than the FT group, with the most pronounced disparity among female students, whose suicide mortality rate in the PT/C group was 43.3 per 100 000 in 2024, over 6-fold higher than in the FT group. Multivariable logistic regression revealed that suicides in the PT/C group had higher odds of individuals aged 19 years or older (adjusted OR [AOR], 26.78 [95% CI, 8.88-116.49]), with a history of psychiatric treatment (AOR, 3.80 [95% CI, 2.65-5.46]), and with previous suicide attempts (AOR, 2.03 [95% CI, 1.42-2.90]) (Table 2). Health-related and other suicide motives were also more common in the PT/C group. After adjusting for all characteristics, the odds that suicide decedents were enrolled in PT/C programs increased from 2022 to 2024 (AOR, 1.87; 95% CI, 1.27-2.77).

**Table 1.** Suicide Mortality by High School Program Type and Sex: Annual and Cumulative Data, 2022-2024

Characteristic	FT program			PT/C program			IRR (95% CI)^b^
	Suicide deaths, No. (%)	Student population	Suicide mortality rate^a^	Suicide deaths, No. (%)	Student population	Suicide mortality rate^a^	
2022-2024							
Overall	680 (100.0)	8 712 804	7.8	328 (100.0)	1 008 050	32.5	4.17 (3.65-4.75)
Male	394 (57.9)	4 446 732	8.9	134 (40.9)	499 385	26.8	3.03 (2.48-3.67)
Female	286 (42.1)	4 266 072	6.7	194 (59.1)	508 665	38.1	5.69 (4.74-6.82)
2022							
Overall	259 (100.0)	2 933 199	8.8	89 (100.0)	310 089	28.7	3.25 (2.54-4.12)
Male	161 (62.2)	1 491 676	10.8	42 (47.2)	157 918	26.6	2.46 (1.73-3.42)
Female	98 (37.8)	1 441 523	6.8	47 (52.8)	152 171	30.9	4.54 (3.18-6.39)
2023							
Overall	214 (100.0)	2 896 238	7.4	114 (100.0)	335 527	34.0	4.60 (3.65-5.76)
Male	123 (57.5)	1 479 814	8.3	48 (42.1)	166 233	28.9	3.47 (2.47-4.81)
Female	91 (42.5)	1 416 424	6.4	66 (57.9)	169 294	39.0	6.07 (4.41-8.31)
2024							
Overall	207 (100.0)	2 883 367	7.2	125 (100.0)	362 434	34.5	4.80 (3.84-5.99)
Male	110 (53.1)	1 475 242	7.5	44 (35.2)	175 234	25.1	3.37 (2.35-4.74)
Female	97 (46.9)	1 408 125	6.9	81 (64.8)	187 200	43.3	6.28 (4.67-8.43)

Abbreviations: FT, full-time; IRR, incidence rate ratio; PT/C, part-time or correspondence.

^a^ Suicide mortality rate was calculated as the number of deaths per 100 000 students. The numbers of students in each program type were extracted from publicly available data in the School Basic Survey (Ministry of Education, Culture, Sports, Science and Technology).

^b^ IRRs represent the relative suicide mortality rate in PT/C programs compared with FT programs. IRRs and 2-sided 95% CIs were estimated using Poisson regression with the profile likelihood method. Estimates were considered statistically significant if their 2-sided 95% CIs excluded 1.00.

**Table 2.** Comparison of Characteristics Between FT and PT/C Students in Suicide Cases: Cumulative Data, 2022-2024

Variable	Students, No. (%)		OR (95% CI)^a^	
	FT program (n = 680)	PT/C program (n = 328)	Unadjusted	Adjusted
Individual characteristics				
Age ≥19 y	3 (0.4)	34 (10.4)	26.10 (9.29-108.99)	26.78 (8.88-116.49)
Male sex	394 (57.9)	134 (40.9)	0.50 (0.38-0.66)	0.87 (0.63-1.21)
Metropolitan residence^b^	205 (30.1)	102 (31.1)	1.05 (0.79-1.39)	0.96 (0.68-1.35)
Living apart from 1 or both parents	224 (32.9)	139 (42.4)	1.50 (1.14-1.96)	1.28 (0.92-1.77)
Suicide attempt history	113 (16.6)	142 (43.3)	3.83 (2.85-5.17)	2.03 (1.42-2.90)
Psychiatric treatment history	139 (20.4)	201 (61.3)	6.16 (4.62-8.25)	3.80 (2.65-5.46)
Causes or motives of suicides^c^^,^^d^				
Family issues	102 (15.0)	44 (13.4)	0.88 (0.60-1.28)	1.19 (0.73-1.93)
Health issues	125 (18.4)	172 (52.4)	4.90 (3.67-6.56)	2.41 (1.52-3.82)
Economic or life issues	8 (1.2)	7 (2.1)	1.83 (0.64-5.15)	1.58 (0.42-5.86)
Relationship issues	58 (8.5)	30 (9.1)	1.08 (0.67-1.70)	1.30 (0.70-2.37)
School-related issues	346 (50.9)	99 (30.2)	0.42 (0.32-0.55)	0.82 (0.54-1.25)
Other issues^e^	65 (9.6)	36 (11.0)	1.17 (0.75-1.78)	2.16 (1.24-3.73)
Unknown	112 (16.5)	42 (12.8)	0.75 (0.50-1.08)	1.65 (0.90-3.02)
Survey year				
2022	259 (38.1)	89 (27.1)	1 [Reference]	1 [Reference]
2023	214 (31.5)	114 (34.8)	1.55 (1.11-2.16)	1.63 (1.10-2.41)
2024	207 (30.4)	125 (38.1)	1.76 (1.27-2.44)	1.87 (1.27-2.77)

Abbreviations: FT, full-time; OR, odds ratio; PT/C, part-time or correspondence.

^a^ ORs and 2-sided 95% CIs were estimated using logistic regression with the profile likelihood method. Adjusted ORs simultaneously controlled for all variables listed. Estimates were considered statistically significant if their 2-sided 95% CIs excluded 1.00.

^b^ Metropolitan residence was defined according to the classification of municipalities by the Statistics Bureau of Japan, which includes designated cities, core cities, and special wards in Tokyo.

^c^ Causes or motives of suicides can be counted up to 4 per suicide. Therefore, the number of specified causes or motives did not necessarily equal the total number of causes or motives.

^d^ Work-related issues were excluded because of the small number of cases (<1% of all suicides).

^e^ Other issues are based on the National Police Agency’s classification, including crime victimization, crime detection, troubles on social media/internet, concerns or discrimination for being a sexual minority, loneliness, relationship with neighbors, suicide following a death, abuse or violence by others (other than family members, roommates, or dating partners), and others.

## Discussion

This study revealed a disparity in suicide risk across high school programs, which was significant in the PT/C group, particularly female students, whose suicide mortality rate increased steadily, reaching 43.3 per 100 000 in 2024, over 6-fold higher than in the FT group. The elevated suicide risk in the PT/C group may reflect a concentration of mental health–related vulnerabilities.^2^ Suicide decedents in the PT/C group tended to have a history of psychiatric treatment or prior suicide attempts, and motives were often attributed to health conditions. The higher proportion of students aged 19 years or older in the PT/C group warrants investigation to disentangle heterogeneity within the group; a prior study reports associations with health risk behaviors, with suicide-related associations attenuating after adjustment for other risk factors.^4^ The adjusted odds in the PT/C group increased over time, suggesting that the increase in suicides is not fully explained by measured characteristics alone. Structural features of PT/C programs—fewer routine interactions with school staff and peers due to part-time and remote instruction—may exacerbate student isolation and hinder early detection of suicide risk.^5^ In this context, recent initiatives, such as mental health e-screening, may strengthen risk identification and sustained engagement in PT/C settings.^6^ This study is limited by reliance solely on suicide statistics, which precludes direct assessment of underlying mechanisms. Overall, these findings underscore the need to prioritize PT/C students, especially female students, by linking early identification with welfare-oriented outreach and support.

## Notes

Supplement.
Data Sharing Statement
