# Supplementary material for: Suicides and High School Program Types in Japan
Source: JAMA Netw Open. 2026 May 27;9(5):e2614997. doi: 10.1001/jamanetworkopen.2026.14997 (PMC13216977; doi:10.1001/jamanetworkopen.2026.14997)
Supplement: Supplement. — Data Sharing Statement [file jamanetwopen-e2614997-s001.pdf]

Data Sharing Statement

Goto. Suicides and High School Program Types in Japan. *JAMA Netw Open*. Published May 27, 2026. doi:10.1001/jamanetworkopen.2026.14997

Data
Data available: No

Additional Information
Explanation for why data not available: The suicide data analyzed in this study were provided by the Ministry of Health, Labour and Welfare (MHLW) based on records aggregated by the National Police Agency (NPA). These data were accessed through an agreement with the MHLW and the NPA, and are not publicly available. The authors are restricted from sharing these data with third parties. Student enrollment data by program type were obtained from the publicly available School Basic Survey (https://www.e-stat.go.jp/).
